# Iodine-complex directed synthesis of PbS quantum dots with enhanced electronic coupling for NIR photodetection

**DOI:** 10.3389/fchem.2025.1677906

**Published:** 2025-10-15

**Authors:** Shenghui He, Guojiang Qian, Cong Zhang, Xingtian Yin, Wenxiu Que

**Affiliations:** Electronic Materials Research Laboratory, Key Laboratory of the Ministry of Education & International Center for Dielectric Research, Shaanxi Engineering Research Center of Advanced Energy Materials and Devices, School of Electronic Science and Engineering, Xi’an Jiaotong University, Xi’an, Shaanxi, China

**Keywords:** PbS QDs, iodine-complex directed synthesis, QDs coupling, photo-FETs, photodiodes

## Abstract

Lead sulfide (PbS) colloidal quantum dots (CQDs) are promising materials for near-infrared (NIR) photodetection. However, conventional synthetic approaches often rely on long-chain organic ligands that impede charge transfer, necessitating complex post-synthetic ligand exchanges. Here, we introduce an Iodine-Complex Directed Synthesis (ICDS) method that enables the direct synthesis of iodide-passivated PbS-I QDs in polar solvents, thereby bypassing traditional hot-injection routes and ligand exchange processes. The PbS-I QDs demonstrated a reduction in interparticle spacing and enhanced electronic coupling, attributable to the elimination of long-chain insulating ligands. Consequently, these PbS-I QDs exhibited a photoluminescence emission peak at 1,060 nm, characterized by a distinct spectral profile indicative of efficient radiative recombination. To assess their practical applicability, the PbS-I QDs were applied in two distinct NIR photodetector architectures: sensitized photo field-effect transistors (photo-FETs) and photodiodes. The photo-FETs have demonstrated a specific detectivity of 1.63 × 10^11^ Jones with rise and decay times recorded at 46.2 ms and 46.3 ms, respectively. In contrast, the photodiodes displayed superior response times, characterized by rise and decay times of 10 μs and 15 μs, respectively. These results demonstrate the effectiveness of the ICDS method in producing high-quality QDs and its potential for enabling high-speed, low-noise NIR photodetectors.

## 1 Introduction

Colloidal quantum dots (CQDs) have emerged as promising materials for next-generation light sensing applications, attributed to their size-tunable bandgaps, high absorption coefficients, and compatibility with solution-based processing (Kwon et al., 2021; Liu et al., 2021; Voznyy et al., 2017; Zheng et al., 2020). Among these materials, lead sulfide (PbS) QDs are particularly distinguished for their efficacy in the near-infrared (NIR) spectrum, making them highly suitable for photodetectors that operate outside the visible light range (Biondi et al., 2021; Ji et al., 2024; Yoo et al., 2021). Traditionally, the synthesis of PbS CQDs is achieved through hot-injection or cation-exchange methods, which afford enhanced control over particle size and colloidal stability. However, these synthesis approaches typically involve the use of long-chain organic ligands, such as oleic acid (OA) or oleylamine (OAm), to stabilize the nanocrystals (Zhang et al., 2023; Zhang et al., 2025). The insulating layers formed by these ligands significantly hinder inter-dot charge transfer, a crucial aspect for optimizing the performance of high-efficiency photodetectors. Post-synthetic ligand exchange strategies aim to replace long ligands with shorter, conductive ones to improve electronic coupling (Baek et al., 2019; Choi et al., 2020; Sun et al., 2020). Solid-state ligand exchange (SSLE) improves film conductivity but often results in film cracking and shrinkage, while solution-phase ligand exchange (SPLE) is scalable yet faces challenges related to material loss and purification (Biondi et al., 2020; Kim et al., 2020). Both methods decouple ligand optimization from the synthesis process, thereby complicating device fabrication and reproducibility.

Direct synthesis of PbS QDs has emerged as a promising alternative to conventional synthetic routes (Moreels et al., 2011; Wang et al., 2019). This approach circumvents the need for traditional hot-injection methods and subsequent ligand exchange processes by enabling the formation of QDs directly in polar solvents, where *in situ* iodide passivation is achieved through iodide species released from precursor complexes (Chen et al., 2023; Liu et al., 2020). Such a strategy not only simplifies processing but also offers significant potential for cost reduction. Ma et al. developed a one-step, scalable synthesis of iodide-passivated PbS QD inks, lowering the material cost to under $6 per gram (Wang et al., 2019). They further introduced a halide-coordination strategy to remove excess PbI_2_, which enhanced inter-dot coupling and diminished trap density, resulting in PCE efficiencies exceeding 12% (Li et al., 2021). Building on this, Liu et al. presented a low-temperature nucleation and high-temperature growth approach, which facilitates meticulous regulation of QD size and distribution. The resulting inks were used in SWIR solar cells with a PCE of 8.79% and EQE up to 70% (Zhou et al., 2025). Although these developments have significantly advanced the use of directly synthesized PbS QDs in solar cells, their investigation within the context of NIR photodetectors remains considerably underexplored (Chuang et al., 2014). The current limitations associated with direct synthesis, including the difficulty in attaining narrower size distributions, reducing aggregation, and enhancing mechanistic insights to enable precise control, are recognized and may adversely affect device performance (Hu et al., 2021; Proppe et al., 2018; Xia et al., 2020). These constraints are especially critical in efforts to achieve ultra-low dark current and elevated specific detectivity (D*). Therefore, a systematic investigation into the performance of directly synthesized PbS QDs within NIR photodetectors is imperative. This exploration will not only address the current research gap but also critically assess how the inherent limitations of the synthesis route impact device metrics crucial for photodetection.

In this work, we propose an Iodine-Complex Directed Synthesis (ICDS) method to comprehensively study the synthesis-structure-performance relationship of PbS-I QDs. We systematically compare their structural, morphological, and optoelectronic properties with those of conventionally synthesized PbS-OA QDs. The results reveal that *in situ* iodide passivation and controlled nucleation inherent to the ICDS reduce interparticle spacing, enhance electronic coupling. When incorporated into NIR photodetectors, these PbS-I QDs enhance the performance of sensitized photo-FETs and photodiodes, delivering high detectivity and rapid response times. These results highlight the efficacy of the ICDS approach in optimizing the optoelectronic properties of QDs during synthesis. Overall, these findings demonstrate the promise of ICDS for producing high-quality QDs suitable for infrared photodetection applications.

## 2 Results and discussion

### 2.1 Synthetic strategies of PbS QDs

**FIGURE 1:**
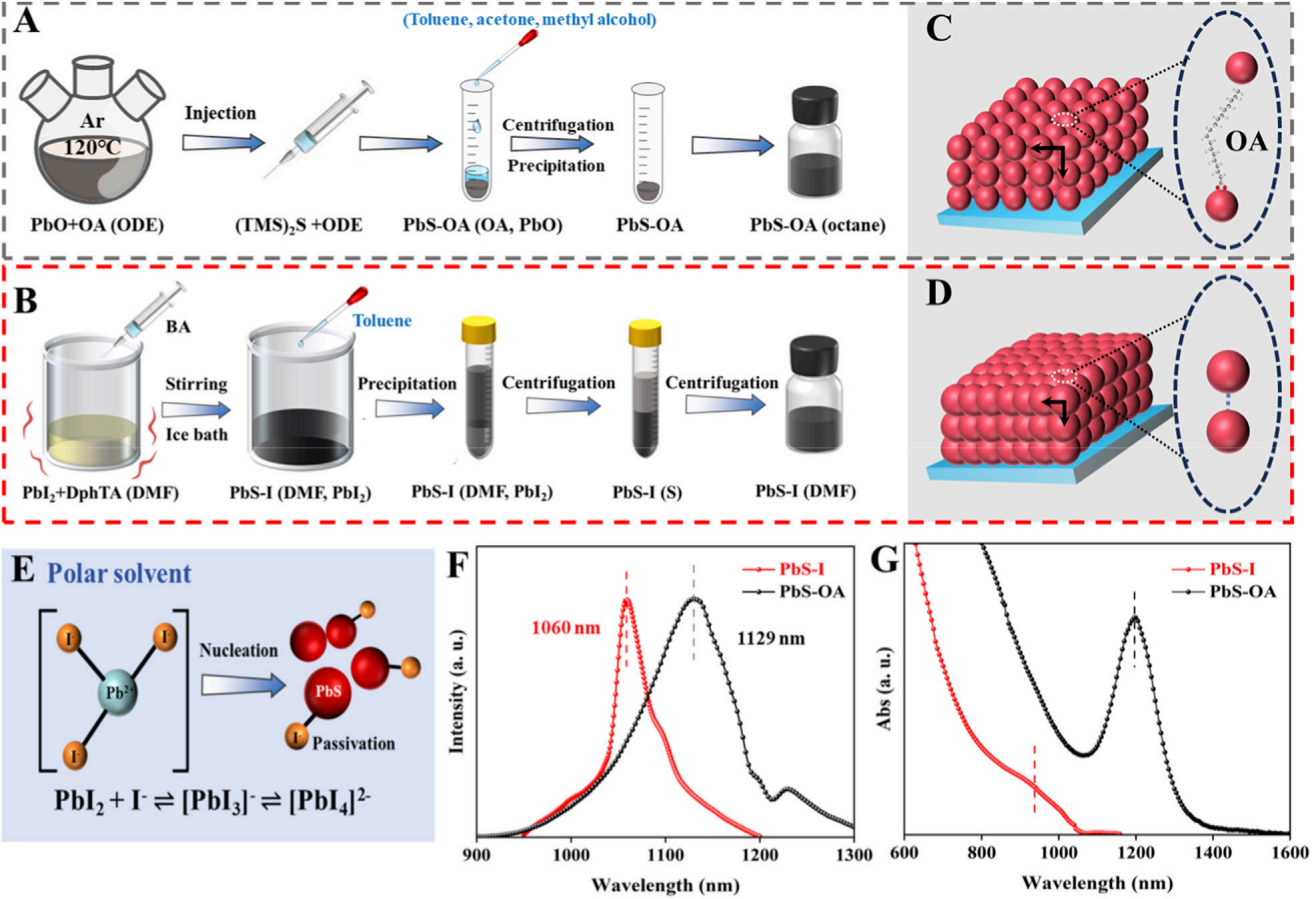
Schematic representation for **(A)** hot-injection synthesis of PbS-OA QDs and **(B)** the direct synthesis of PbS-I QDs. **(C,D)** Schematic illustration of QD matrix arrangement under two different synthesis methods. **(E)** Schematic depiction of the translation between different iodine-complexes. **(F)** PL and **(G)** Abs spectra of PbS-OA QDs and PbS-I QDs.

To clarify the fundamental differences in synthetic methodologies and their influence on the properties of QDs, Figure 1 presents a systematic comparison of the conventional hot-injection (Figure 1A) and the innovative direct synthesis methods (Figure 1B) for PbS QDs. In the hot-injection process, the Pb precursor (PbO) is solubilized in oleic acid (OA) within a nonpolar solvent, such as 1-octadecene (ODE), and is maintained under inert conditions at an elevated temperature of 120 °C (Biondi et al., 2021). The procedure is initiated by the rapid introduction of a sulfur source, typically bis(trimethylsilyl)sulfide (TMS_2_S), which instigates nucleation followed by growth under high-temperature conditions. This method produces highly monodisperse PbS QDs that are stabilized with long-chain insulating oleic acid (OA) ligands, referred to as PbS-OA. In contrast, the direct synthesis approach avoids the need for high-temperature injection and ligand exchange by employing polar solvents, such as dimethylformamide (DMF), in conjunction with reactive iodine-coordinated lead complexes (PbI_2_ + DphTA) to facilitate nucleation at low temperatures. This process is followed by *in situ* ligand passivation using iodide. The resultant QDs, referred to as PbS-I, are produced directly in colloidal form, eliminating the necessity for additional ligand exchange. Figures 1C,D depict the divergent assembly behaviors that emerge from the two distinct synthesis methods. The PbS-OA QDs illustrated in Figure 1C are hindered by the presence of bulky, insulating OA ligands, resulting in a loosely packed structure and increased inter-dot distances. This spatial separation restricts charge transfer due to the diminished electronic coupling between the QDs (Li et al., 2016). Conversely, as illustrated in Figure 1D, the PbS-I QDs exhibit significantly reduced interparticle spacing, which can be ascribed to the utilization of short, ionic passivating ligands, specifically iodide ions. The resultant compact and ordered matrix is anticipated to enhance electronic coupling and facilitate carrier transfer throughout the film. The schematic presented in Figure 1D highlights the significant role of iodine-complexes ([PbI_3_]^-^ and [PbI_4_]^2-^) in the regulation of nucleation (Chen et al., 2024; Tang et al., 2019). These polyiodide complexes are formed dynamically in the presence of polar solvents such as dimethylformamide (DMF) or butylamine, which modulate the availability of free Pb^2+^ ions and facilitate the *in situ* passivation of QD surfaces (Green et al., 2019). This method to passivation in solution enhances the stability of the QDs and ensures their compatibility with subsequent solution processing, potentially obviating the necessity for ex-ligand exchange. Furthermore, the reversible equilibrium among PbI_2_, I^−^, and [PbI_x_]^n−^ intermediates plays a critical role in influencing the nucleation rate Figure 1E. In contrast to the hot-injection method, which facilitates immediate nucleation through the swift availability of precursors, the direct synthesis approach allows for a more controlled and gradual release of Pb^2+^ and I^−^ ions through complexation (Zhou et al., 2025). Specifically, in polar solvents PbI_2_ and I^−^ form equilibria between [PbI_3_]^−^ and [PbI_4_]^2−^, as described by the following Equations 1 and 2:

$${\text{PbI}}_{2}+I^{-}⇌{\left[{\text{PbI}}_{3}\right]}^{-}$$ (1)

$${\left[{\text{PbI}}_{3}\right]}^{-}+I^{-}⇌{\left[{\text{PbI}}_{4}\right]}^{2-}$$ (2)

and the balance between these species critically governs the activity of reactive Pb(II). A [PbI_3_]^−^-rich environment more readily releases Pb(II), leading to shorter induction times and higher nucleus density, whereas a [PbI_4_]^2−^-rich environment stabilizes Pb(II), depresses effective monomer supersaturation, and thus delays nucleation. This framework directly links iodoplumbate equilibria to the LaMer model of burst nucleation and provides a rational basis for the controllability of ICDS (Liu et al., 2021; Vreeland et al., 2015).

### 2.2 Optical characteristics of PbS-I QDs

The photoluminescence (PL) and absorption (Abs) spectra presented in Figures 1F,G show the optical characteristics of the synthesized QDs. The PL spectrum of PbS-I QDs exhibits an emission peak centered at 1,060 nm with a narrower profile, while the PbS-OA QDs show a peak at 1,129 nm with a broader emission. As shown in the Abs spectra, PbS-I QDs display a broad and featureless profile, while PbS-OA QDs exhibit a more distinct absorption peak located at 1,165 nm (Tumen-Ulzii et al., 2020). Additionally, the effects of reaction temperature, reaction time on the nucleation and growth of QDs are also investigated. Supplementary Figure S1A demonstrates that increasing the reaction temperature from 0 °C to 75 °C enhances the crystallinity of PbS-I QDs, as evidenced by the increased intensity and sharpening of the XRD peaks. In contrast, extending the reaction time at 25 °C (Supplementary Figure S1B) results in only a slight improvement in crystal quality. PL spectra (Supplementary Figures S2A,C) show only a slight change in PL intensity with increasing temperature and longer reaction times, indicating limited enhancement in crystallinity. Additionally, the full width at half maximum of the PL peak broadens with both higher temperatures and extended reaction durations, suggesting a widening of the size distribution under these conditions. Notably, the observed redshift in the absorption peaks (Supplementary Figures S2B,D) with increasing reaction temperature and extended reaction time suggests an increase in the average size of the PbS-I QDs is occurs because higher temperatures and longer durations facilitate crystal growth and coalescence, reducing the bandgap energy and thus weakening the quantum confinement effect. Overall, these results indicate that the ICDS method enables the preparation of high-quality QDs under low-temperature conditions, eliminating the need for high-temperature synthesis to achieve required crystallinity and optical properties.

### 2.3 Morphology and surface chemistry of PbS-I QDs

**FIGURE 2:**
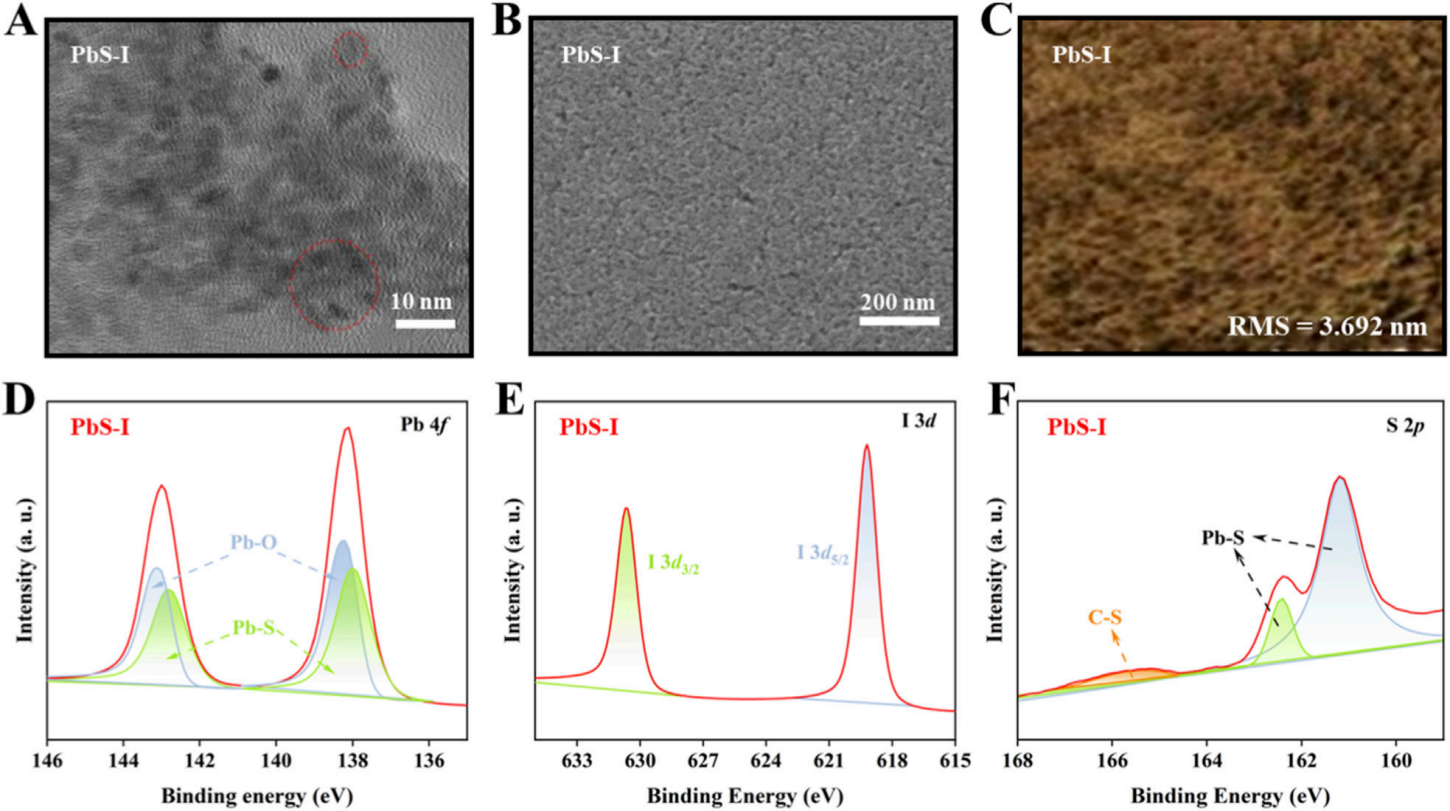
**(A)** TEM images of PbS-I QDs, **(B)** SEM, **(C)** AFM images of PbS-I QD film. XPS core-level spectra of **(D)** Pb 4*f*, **(E)** I 3*d*, and **(F)** S 2*p* of PbS-I QDs.

Further investigates the morphological, structural, and surface chemical properties of PbS-I QDs synthesized through direct synthesis. Figure 2A presents a transmission electron microscopy (TEM) image of the PbS-I QDs, showing nanoparticles with well-defined morphology. Although individual QDs are distinguishable in certain regions, the image also shows partial aggregation and interparticle crosslinking (red areas). Furthermore, TEM of low-density dispersed particles in Supplementary Figure S3 reveals that the size of PbS QDs synthesized via the ICDS method is approximately 3.9 nm. However, most nanocrystals form clusters with sizes close to 9 nm due to interparticle crosslinking, indicating the limited colloidal stability of the directly synthesized QDs. This morphological feature directly correlates with the reduced absorption peak intensity, as aggregated QDs enhance light scattering and non-radiative energy losses (Manser et al., 2016). Furthermore, the scanning electron microscopy (SEM) image (Figure 2B) shows a relatively compact PbS-I QD film with only minor cracks observed in certain regions, suggesting generally favorable film-forming characteristics. In conjunction with this, the atomic force microscopy (AFM) image presented in Figure 2C reveals a relatively smooth surface, with a root-mean-square (RMS) roughness of 3.692 nm. This smooth morphology is beneficial for device applications, as it enhances charge transfer between layers. Figures 2D–F illustrate the core-level spectra obtained from X-ray photoelectron spectroscopy (XPS), which elucidate the surface chemical environment of PbS-I QDs. As shown in Figure 2D, the Pb 4*f* spectrum reveals two prominent peaks at approximately 138.6 eV and 143.5 eV, corresponding to Pb 4*f*_7/2_ and Pb 4*f*_5/2_, respectively. These peaks are characteristic of Pb in PbS-I QDs (Ding et al., 2022; Xia et al., 2019; Xue et al., 2019). The deconvolution of the spectrum reveals minor contributions from Pb-O bonds, likely due to minimal surface oxidation. However, the relatively low intensity of this oxide component is a critical observation, as it indicates that iodide passivation effectively suppresses surface oxidation. The formation of stable Pb-I bonds passivates the otherwise highly reactive surface Pb sites, protecting them from reaction with ambient oxygen (Ding et al., 2025). The I 3*d* (Figure 2E) spectrum presented peaks at 619.2 eV and 630.7 eV, corresponding to I 3*d*_5/2_ and I 3*d*_3/2_, which are characteristic of surface-bound iodide species rather than free iodide salts, confirming the presence of I^−^ on the QD surface (Zhang et al., 2024). Meanwhile, the symmetric nature and narrow full-width at half maximum (FWHM) of these peaks suggest a well-defined chemical environment for iodide ions. The presence of these features confirms the successful incorporation of iodide species on the QD surface and supports the formation of [PbI_x_]^n−^ complexes (e.g [PbI_3_]^−^ and [PbI_4_]^2−^), which are known to facilitate both nucleation and *in situ* passivation. Figure 2F displays the S 2*p* spectrum with characteristic S 2*p*_3_/_2_ and S 2*p*_1_/_2_ peaks at 161.5 eV and 162.8 eV, corresponding to Pb-S bonding in the crystalline core. A minor peak at 168.5 eV is also observed, which is attributed to C-S bonding, possibly resulting from residual organic species (Zhang et al., 2024). However, its relative area constitutes less than 6.3% of the total S signal, confirming that the QD surface is primarily passivated by inorganic iodide rather than long-chain organic ligands. This inorganic-dominated surface passivation is essential for promoting efficient electronic coupling between QDs. In summary, these results demonstrate that the direct synthesis strategy produces PbS-I quantum dots with superior crystalline quality, uniform morphology, and effective surface passivation via iodide ligands. The minimized surface oxidation and reduced organic residue contribute to improved electronic coupling and charge transfer in quantum dot-based optoelectronic devices. These findings highlight the potential of iodine-complex-mediated direct synthesis as a powerful method for producing high-performance QDs.

### 2.4 Sensitized photo-FET based on PbS-I QDs

**FIGURE 3:**
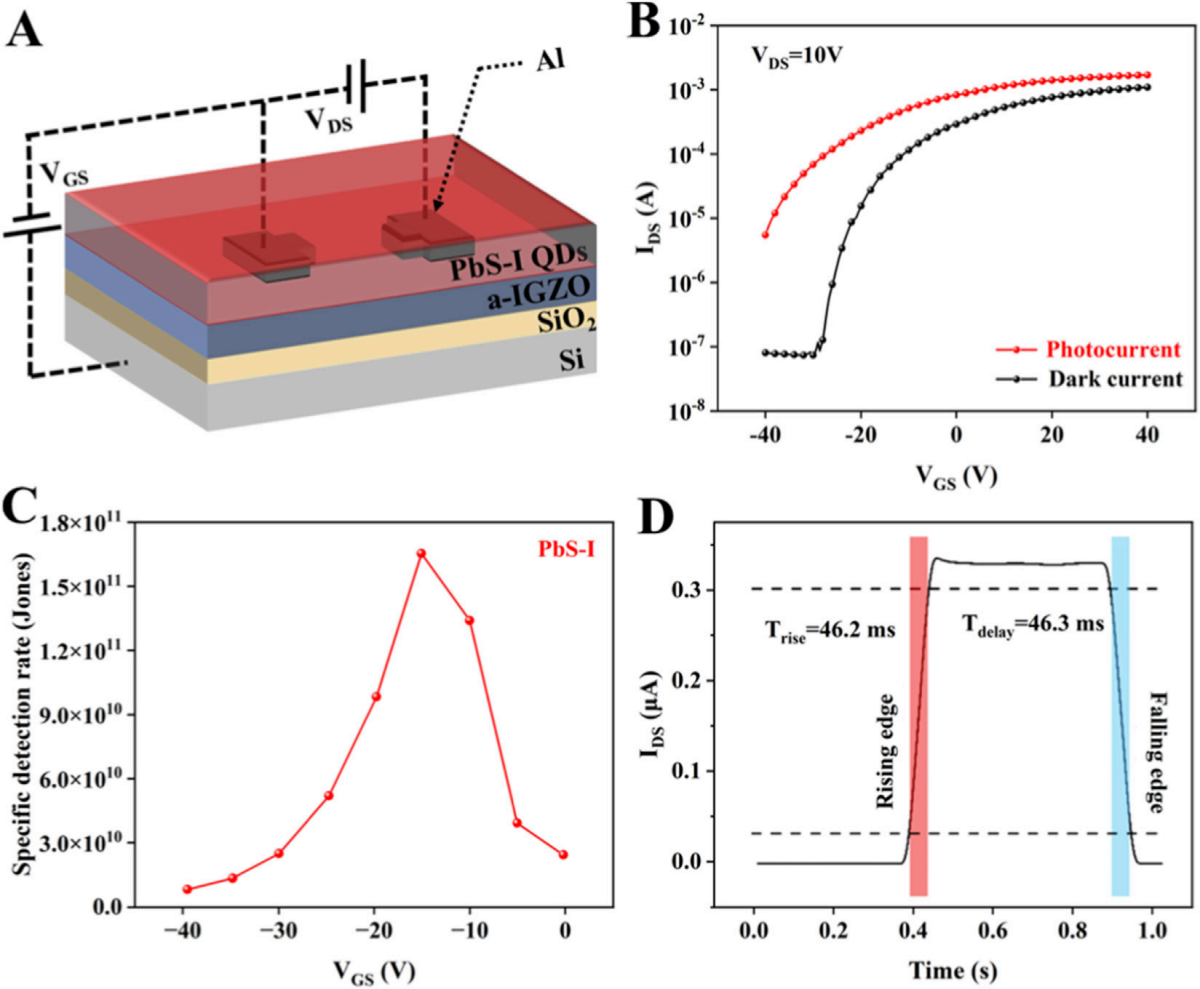
**(A)** Schematic structure of the sensitized Photo-FET. **(B)** Transfer characteristics of the PbS-I based sensitized Photo-FETs under dark and NIR illumination. **(C)** Gate-voltage dependent specific D* of the device. **(D)** The transient response curves of the PbS-I based sensitized Photo-FETs.

To further examine the practical applicability of directly synthesized PbS-I QDs in optoelectronic devices, we investigate integration into a sensitized NIR photodetector utilizing a Photo-FET architecture (Figure 3A). While prior research has established the structural uniformity, surface passivation, and optical advantages of PbS-I QDs, this investigation shifts its focus toward translating these attributes into enhanced device performance. By incorporating PbS-I QDs as a photosensitizing layer on an indium gallium zinc oxide (IGZO) semiconductor channel, the device leverages the strong NIR absorption and efficient charge separation properties of the QDs, alongside the high mobility and low dark current characteristics of IGZO (García de Arquer et al., 2021). This hybrid configuration offers a promising framework for developing photodetectors that demonstrate both high detectivity and rapid response times, while remaining fully compatible with solution-processable fabrication techniques. As illustrated in Figure 3B, the transfer characteristics (I_DS_-V_GS_) of the device are analyzed under both dark and illuminated conditions at a V_DS_ of 10 V. A significant enhancement in I_DS_ is noted under NIR illumination throughout the entire range of gate voltages, indicating effective photogating behavior. Under near-infrared illumination, the transfer curve exhibits a more gradual subthreshold region compared to the dark state, which can be attributed to the increased carrier density. Despite the slight broadening of the subthreshold slope, the overall drain current significantly increases, confirming enhanced photoconductivity and efficient photogating behavior (Ling et al., 2020). This trade-off between subthreshold modulation and photocurrent generation underscores the improved photoresponsivity of the device, making it suited for NIR photodetection applications. Although the dark current remains as low as 8.8 × 10^−8^ A at a V_GS_ of −40 V, the device exhibits the maximum SNR of 9.7 × 10^2^ at V_GS_ = −30 V, corresponding to a photocurrent of 5.9 × 10^−4^ A. As illustrated in Figure 3C, the detectivity (D*) exhibits a strong dependence on V_GS_, underscoring the critical role of electrostatic gating in modulating carrier separation efficiency. The specific D* is defined by Equation 3 (Sun et al., 2021):

$$D^{*}=\frac{R}{\sqrt{2qI_{dark}}}$$ (3)

where *R* is the responsivity, *q* is the elementary charge, and *I*_*dark*_ is the dark current (Sun et al., 2021). At V_GS_ = −15 V under 1,064 nm illumination (90.6 mW/cm^2^), the device achieves a peak detectivity of 1.63 × 10^11^ Jones, demonstrating its potential for NIR photodetection applications based on directly synthesized PbS-I QDs. Figure 3D presents the transient response characteristics of the optimized device. The rise time (T_rise_ = 46.2 ms) and fall time (T_delay_ = 46.3 ms) exhibit a high degree of symmetry, which signifies rapid and reversible response dynamics. These temporal parameters are predominantly constrained by the rates of interfacial charge transfer and the kinetics of recombination at the QDs/IGZO interface (Choi et al., 2020). The nearly identical rise and decay times indicate that the device exhibits fast and fully reversible photo-response behavior upon light on/off switching. This dynamic response reflects efficient interfacial charge transfer and minimal carrier trapping at the QDs/IGZO interface (Zhang et al., 2024). These performance benefits can be directly attributed to the direct synthesis strategy, which enables the *in situ* incorporation of short, ionic iodide ligands during QD formation. This approach minimizes interdot spacing and enhances electronic coupling between neighboring QDs, thereby facilitating exciton dissociation and interfacial charge transfer.

### 2.5 Photodiode based on PbS-I QDs

**FIGURE 4:**
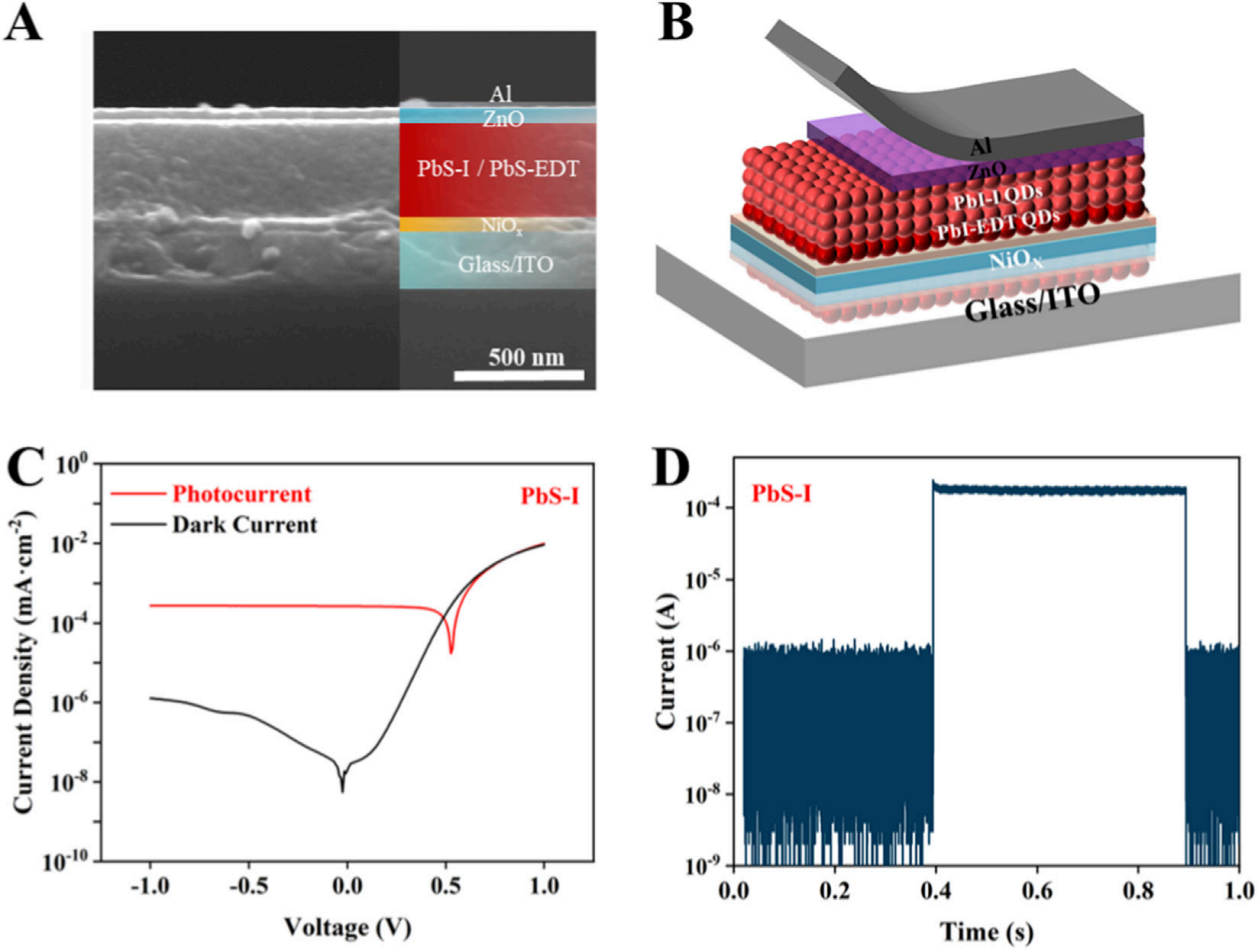
**(A)** Cross-sectional SEM image of PbS-I QD photodiode. **(B)** Schematic diagram of photodiode architecture. **(C)** I–V curves under dark and NIR light of the PbS-I QD device. **(D)** Transient response curves of the PbS-I QD device.

In order to evaluate the performance limits and application-specific advantages of directly synthesized PbS-I QDs, we further investigate the incorporation of PbS-I QDs into a vertical photodiode architecture. This configuration offers significant benefits compared to the previously analyzed phototransistor setup. While sensitized photo-FETs exhibit high gain and low dark current, their response times, which are on the order of milliseconds, make them more suitable for static or low-speed imaging applications (Kim et al., 2020; Liu et al., 2017). The cross-sectional SEM (Figure 4A) image confirms the successful construction of a multilayered vertical device, which consists of the following layers: ITO/Glass/NiOx/PbS-EDT QDs/PbS-I QDs/ZnO/Al (Du et al., 2018). This structure is illustrated schematically in Figure 4B, which emphasizes the symmetric pathways for carrier extraction that minimize charge recombination and enhance the transient response. Figure 4C illustrates the current-voltage (I–V) of the device when subjected to both dark conditions and 1,064 nm NIR illumination. In comparison to the sensitized photo-FETs, the vertical diode device shows a markedly reduced steady-state dark current, despite utilizing the same PbS-I QDs active layer. Meanwhile, the PbS-I QD photodiode achieves a maximum D* of 1.1 × 10^11^ Jones, with a corresponding responsivity of 0.21 A/W under an incident power density of 90.6 mW/cm^2^, demonstrating its potential for efficient photocarrier extraction. This improvement can be largely attributed to the beneficial intrinsic electric field inherent in the diode structure, which promotes carrier extraction without the requirement for an external gate bias (Li et al., 2021; Liu et al., 2023). The efficacy of the photodiode is significantly influenced by the defect density present in the PbS-I QD film. While PbS-I QDs exhibit remarkable colloidal stability and robust electronic coupling, the existence of intrinsic surface and interfacial trap states, which are further aggravated during the film formation process via spin coating, hinders the suppression of dark current and diminishes the overall responsivity of the device. In sensitized photo-FETs, the presence of defects frequently results in an increase in off-state current by two to three orders of magnitude, thereby undermining the benefits associated with gate-controlled conduction. Conversely, the vertical diode architecture capitalizes on spatial charge separation and unipolar transfer, which enhances its resilience to moderate defect densities. The fast transient response shown in Figure 4D further reinforces the potential of this photodiode structure for optoelectronic applications. The on-off current switching exhibits a nearly rectangular shape with negligible lag or overshoot, indicating rapid carrier separation and extraction (Li et al., 2021). The rise time is 10 μs, and the fall time is approximately 15 μs, which are over three orders of magnitude faster than the 46.2 ms (rise time) and 46.3 ms (fall times) observed in the sensitized photo-FETs (Supplementary Figure S4). This enhancement in speed is attributed to the vertical electric field driving mechanism in the diode, which bypasses the slower drift-diffusion dynamics and interfacial trapping processes typically found in field-effect transfer (Xu et al., 2020). As summarized in Supplementary Table S1, the performance of our photodetector based on ICDS-synthesized PbS-I QDs is competitive with state-of-the-art devices fabricated from conventional QDs that have undergone intensive post-synthetic processing (e.g., multi-step ligand exchanges, delicate solid-state treatments). Crucially, this high performance was attained through a drastically simplified fabrication process of direct synthesis of QDs in polar solvents without the need for complex and wasteful ligand exchange steps. This demonstrates not only the viability but also the significant practical potential of the ICDS approach for developing high-performance, low-cost, and environmentally friendly optoelectronic devices.

These results substantiate that PbS-I QDs synthesized via the ICDS method, when implemented in a vertically structured photodiode with optimized thermal processing, provide an effective balance among fabrication simplicity, material compatibility, and optoelectronic performance. Crucially, this highlights the broad applicability of the direct synthesis route, extending beyond surface-anchored QD phototransistors to scalable photodiodes.

## 3 Conclusion

In summary, we have developed an Iodine-Complex Directed Synthesis method for the direct preparation of iodide-passivated PbS-I QDs in polar solvents. This approach has eliminated the need for conventional hot-injection synthesis and subsequent ligand exchange, offering a simplified and scalable route to high-quality colloidal QDs. Compared to PbS-OA QDs capped with oleic acid, the PbS-I QDs synthesized via this approach exhibit reduced interparticle spacing due to the absence of long-chain insulating ligands, which facilitates improved electronic coupling. To assess their practical applicability, PbS-I QDs synthesized through the ICDS have been incorporated into two distinct architectures for NIR photodetectors: sensitized photo-FETs and photodiodes. The photo-FETs have demonstrated a specific detectivity of 1.63 × 10^11^ Jones and a responsivity of 0.203 A/W, with rise and decay times recorded at 46.2 ms and 46.3 ms, respectively. In contrast, the photodiode configuration has exhibited an improved response speed, achieving a specific detectivity of 1.12 × 10^11^ Jones, alongside a rise time of 10 μs and a decay time of 15 μs. Further exploration of the growth mechanisms and interface engineering in ICDS has the potential to unlock additional performance improvements and broader application scopes.

## Data Availability

The original contributions presented in the study are included in the article/Supplementary Material, further inquiries can be directed to the corresponding author.
