# Supplementary material for: Iodine-complex directed synthesis of PbS quantum dots with enhanced electronic coupling for NIR photodetection
Source: Front Chem. 2025 Oct 15;13:1677906. doi: 10.3389/fchem.2025.1677906 (PMC12568700; doi:10.3389/fchem.2025.1677906)
Supplement: Supplementary file 1 [file DataSheet1.docx]

Supporting Information

**Iodine-Complex Directed Synthesis of PbS Quantum Dots with Enhanced Electronic Coupling for NIR Photodetection**

Shenghui He^1†^, Guojiang Qian^1†^, Cong Zhang^1^, Xingtian Yin^1^* and Wenxiu Que^1^

^1^Electronic Materials Research Laboratory, Key Laboratory of the Ministry of Education & International Center for Dielectric Research, Shaanxi Engineering Research Center of Advanced Energy Materials and Devices, School of Electronic Science and Engineering, Xi’an Jiaotong University, Xi'an 710049, Shaanxi, P. R. China

*Corresponding authors: xt_yin@mail.xjtu.edu.cn (X. Yin)

First authorship: Shenghui He^†^ and Guojiang Qian^†^ These authors contributed equally to this work and share first authorship.

**Supporting Information Contents**

**Supplementary Figures**

**FIGURE S1.** XRD patterns of PbS-I QDs synthesized under different reaction temperatures and different reaction time.

**FIGURE S2.** PL and Abs spectra of PbS-I QDs synthesized under (a) different reaction temperatures and different reaction time.

**FIGURE S3.** TEM images of PbS-I QDs with a low density of particles.

**FIGURE S4.** Transient response of the photodiode under 1 Hz illumination: magnified rise time and magnified decay time.

## Experimental Section

***Chemicals:*** The following chemicals were purchased: lead(II) iodide (PbI_2_, ≥ 99.9%, Xi’an Polymer Light Technology Inc.), lead oxide (PbO, ≥ 99.9%, Aladdin), oleic acid (≥ 99.0%, Aladdin), acetonitrile (ACN, ≥99.9%, Aladdin), butyl amine (C_4_H_11_N, 99%, Innochem), toluene (≥ 99.5%, Sinopharm Chemical Reagent Co., Ltd.), n-hexane (≥ 99.0 %, Aladdin), methyl alcohol (≥99.0%, Aladdin), 1-octadecene (ODE, 90.0%, Aladdin), methyl acetate (MeOAc, 99.0%, Aladdin), octane (Anhydrous, 96%, Shanghai Boer Chemical Reagent Co., Ltd.), hexamethyldisilathiane (95%, Shanghai Boer Chemical Reagent Co., Ltd.), N,N-Dimethylformamide (DMF, ≥99.5%, Shanghai Chengfan Pharmaceutical Co. Ltd.), diphenylthiourea (DphTA, 98%, Beijing Kaiguo Technology Co., Ltd.) , dithioglycol (98%, Beijing Kaiguo Technology Co., Ltd.), NiO ceramic sputtering target (99.99%, HZNM), IGZO ceramic sputtering target (99.99%, HZNM).

***Direct synthesis of PbS-I QD inks:*** For a typical synthesis, 0.06-6 mmol PbI_2_ mmol and 1 mL butylamine (BA) were dissolved in 8 mL DMF with stirring under nitrogen. Before injection of the DPhTA solution, the Pb solution temperature was adjusted according to the desired QD size. When the temperature stabilized, 0.01-1 mmol DPhTA in 1 mL of DMF was rapidly injected into the Pb solution. The molar ratio of PbI_2_/DPhTA keeps 6 for all reactions. The growth time of QDs dependents on the reaction temperatures and concentrations. After reaction, the QDs were isolated by precipitation with toluene as anti-solvent and centrifugation at 8000 rpm for 5 min. The obtained solids were dissolved dissolved in DMF for device fabrication.

***Synthesis of PbS-OA QDs:*** Lead oxide (PbO, 2 mmol, 0.4464 g), octadecene (ODE, 10 ml) and oleic acid (OA, 1.5 ml) were added in a three-neck flask, stirred and vacuumed for 20 mins. Then, the reaction system was heated to 95 °C under negative pressure, and maintained for 3 h. After that, the system was pumped with nitrogen and heated to 120 °C. The mixture of bis(trimethylsilyl)sulfide ((TMS)_2_S, 2 mmol, 210 μL) and octadecene (ODE, 10 ml) was injected into the reaction system swiftly. And the reaction system was reduced to room temperature by means of ice bath. The reaction product was transferred to a separating funnel and layered by adding 25 ml acetone, 5 ml toluene and 10 ml methanol, and the supernatant was removed. Further purification was carried by washing using acetone and toluene for three times. Finally, the product was dried in a vacuum oven to obtain quantum dot power.

Solution-Phase Ligand Exchange Process: Dissolve 0.1 M lead iodide (PbI_2_) and 0.06 M ammonium acetate in DMF solution. Dissolve the oleic acid capped PbS QDs in n-hexane at a concentration of 10 mg·ml^-1^. Mix the above two solutions and shake them violently until they were completely layered. Remove the supernatant through the separating funnel and wash repeatedly with n-hexane to remove free oleic acid. QDs were precipitated by adding toluene of equal volume. Centrifuge to remove supernatant and dry the product in a vacuum oven.

***Sensitized Photo-FETs Fabrication:*** Si/SiO_2_ substrates (the thickness of SiO_2_ was 100 nm) were sequentially cleaned with acetone and anhydrous alcohol in an ultrasonic bath each for 5 mins, followed by an UV-ozone treatment for 15 mins. Then, a layer of a-IGZO film was deposited by RF magnetron sputtering system. In the deposition process, the element ratio of a-IGZO target was In: Ga: Zn = 1: 1: 1, the atmosphere required for deposition was pure argon, the sputtering power was 60 W, and the sputtering time was 5 mins. The a-IGZO films were placed on a hot plate at 400 °C and annealed in air for 60 mins. Subsequently, patterned aluminum electrodes were deposited on a-IGZO film by thermal evaporation with a metal mask. The deposition of the photosensitive layer was completed by spin-coating. PbS-I powder was dissolved in a mixture of butamine/pentamine/hexamine (10:3:2, vol%) at a concentration of 50 mg ml^-1^. Ultrasound the above solution and filter it. Under ambient atmosphere, PbS-I QDs was spin-coated on the substrate at 2500 rpm for 60 s.

***Photodiodes Fabrication:*** ITO substrates (20 mm × 20 mm) were cleaned using detergent, deionized water, acetone, and isopropanol, followed by UV-ozone treatment for 15 min. The NiO_x_ hole transport layer was deposited by RF magnetron sputtering using a 54 mm × 4 mm NiO_x_ ceramic target, with Ar and O_2_ under 90 W power at room temperature. PbS-EDT films were prepared via solid-state ligand exchange (SSLE). PbS-OA QDs in octane were spin-coated onto NiO_x_/ITO, followed by treatment with 0.02 vol% EDT in acetonitrile, rinsing, and repetition to reach the desired thickness. PbS-I absorber layers were formed by spin-coating pre-dispersed PbS-I QDs in organic solvent onto PbS-EDT films, followed by annealing at 70 °C for 10 min. ZnO electron transport layers were synthesized by solution-phase reaction of zinc acetate and KOH in methanol. After purification, ZnO nanoparticles were dispersed (10 mg/mL) and spin-coated onto PbS-I films, then annealed at 70 °C for 30 min. Al top electrodes were deposited via thermal evaporation under high vacuum (6.0 × 10^-4^ Pa), with a deposition rate of ~4 Å/s.

***Characterization:*** XRD analysis was employed to characterize the crystalline properties by using a D/max-2400 XRD spectrometer (Rigaku, Japan) with Cu Ka radiation. The absorption spectra of the samples were obtained by a PE Lambda950 UV-vis-NIR spectrometer. SEM (Quanta 250 FEG, FEI Inc, Hongkong) and AFM used to characterize the surface morphology of films. AFM was carried out with an Innova AFM (Santa Barbara, CA USA) in tapping mode. A transmission electron microscope (TEM, Talos F200X, Thermo Fisher Scientific Inc., USA) was employed to observe the microstructural properties of the prepared QDs. X-ray photoelectron spectroscopy (XPS) was measured on an ESCALAB Xi^+^ system (Thermo Fisher, USA). A transient steady-state fluorescence spectrometer (Edinburgh FLS980, EI, UK) was adopted to determine the steady PL spectra. The Device performance test and SCLC test were completed by the precision source/measure unit (KEYSIGHT, USA), and the light source was provided by monochrome laser of MIL-III-1064-50 mW (Changchun New Industries Optoelectronics Technology Co., Ltd., China). The noise power spectral densities were measured using a semiconductor parameter analyzer (PDA FS380 Pro, Platform Design Automation) at room temperature.

**Formula Section:** The responsivity R (A W^-1^) is defined as the ratio of the photocurrent *I_photo_* (photogenerated current, *I_photo_*=*I_light_*-*I_dark_*) of the device to the incident illumination power *P_in_*:

$\begin{aligned} R=\frac{I_{photo}}{P_{in}}=\frac{I_{light}-I_{dark}}{P_{in}} Eq. S1 \end{aligned}$

The Detectivity D^*^ (cm Hz^1/2^ W^-1^) is calculated based on the noise equivalent power (NEP, W Hz^1/2^) and the square root of active area (A).

$$\begin{aligned} D^{*}=\frac{\sqrt{A}}{NEP}=\frac{R\sqrt{A\Delta f}}{\sqrt{\int_{0}^{\Delta f} S\left( f \right)df}} \#Eq.S2 \end{aligned}$$

The NEP is defined as the input signal power at which the signal-to-noise ratio is 1 and the output bandwidth is 1 Hz.

$$\begin{aligned} NEP=\frac{\sqrt{\frac{\left\langle i_{n}^{2} \right\rangle}{\Deltaƒ}}}{R} \#Eq.S3 \end{aligned}$$

Where Δƒ is the measurement bandwidth over which the noise is considered. The noise current is calculated using following formula:

$$\begin{aligned} \left\langle i_{n}^{2} \right\rangle=\int S\left( f \right)df \#Eq.S4 \end{aligned}$$

Where S(ƒ) is the spectral noise density which measured under dark conditions with respect to the noise frequency ƒ.

The rise time *τ_rise_* and decay time *τ_decay_* are defined as the time required to rise from 10% to 90% of the photocurrent and to decay from 90% to 10%, respectively.

***Statistical Analysis:*** The sample size (n) was described within the captions of the corresponding figures. The Origin Pro Software was used for data processing and analysis.

**Supplementary Figures**


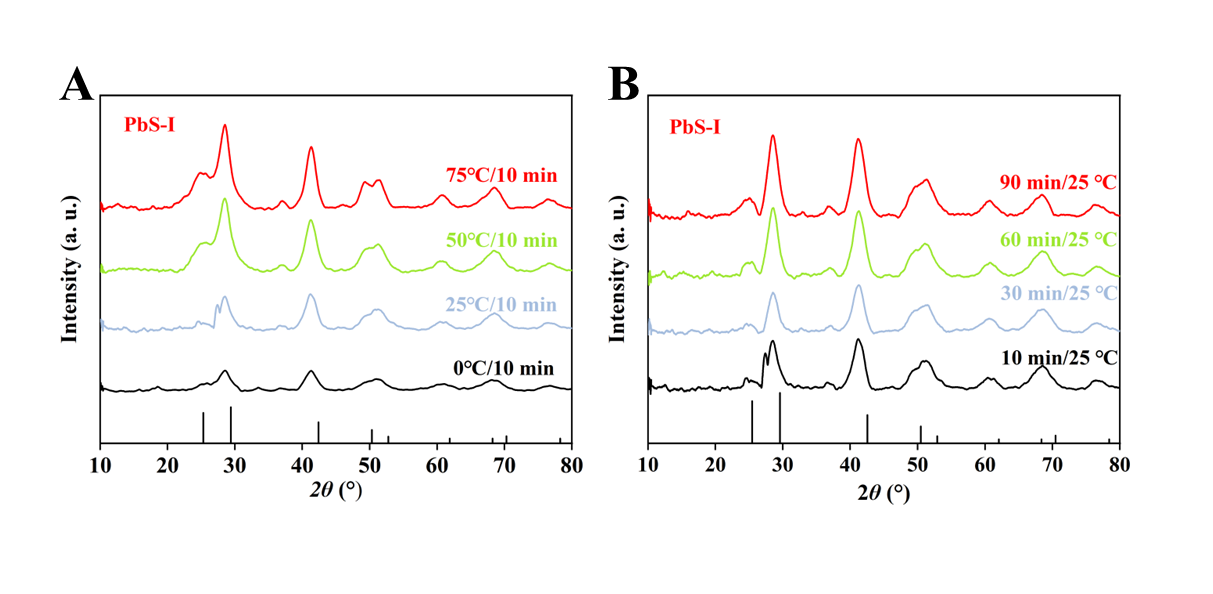


**FIGURE S1** XRD patterns of PbS-I QDs synthesized under (A) different reaction temperatures and (B) different reaction time.


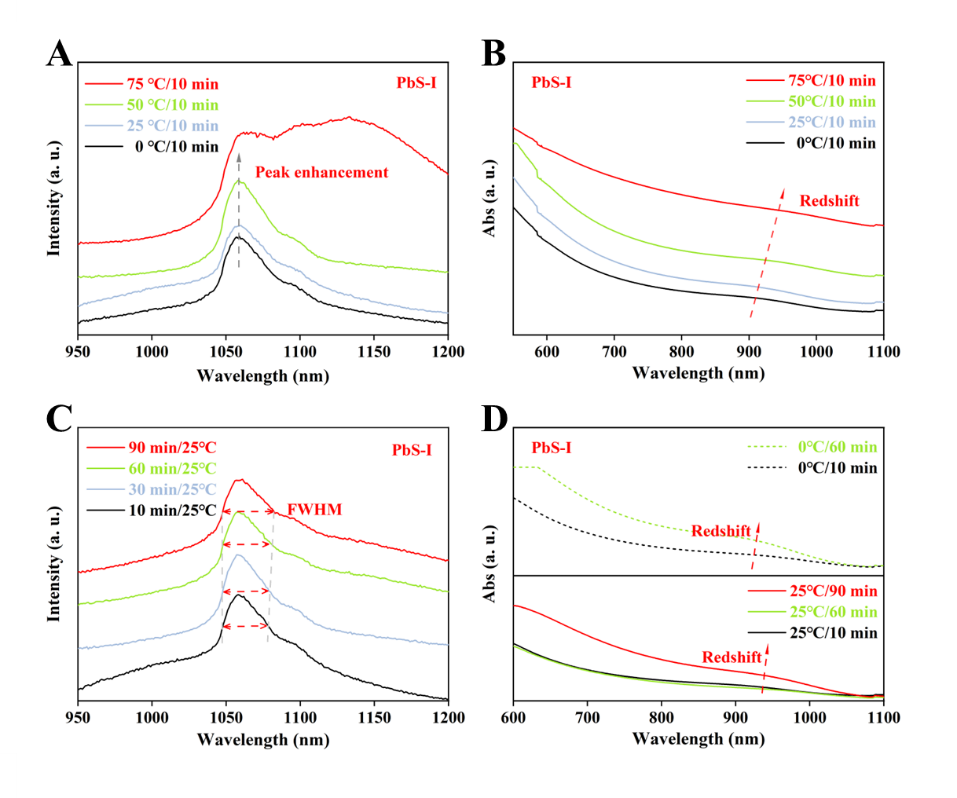


**FIGURE S2** (A) PL and (B) Abs spectra of PbS-I QDs synthesized under different reaction temperatures. (C) PL and (D) Abs spectra of PbS-I QDs synthesized under different reaction time.


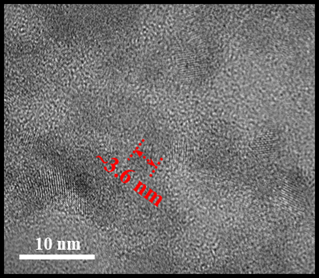


**FIGURE S3** TEM images of PbS-I QDs with a low density of particles.

**
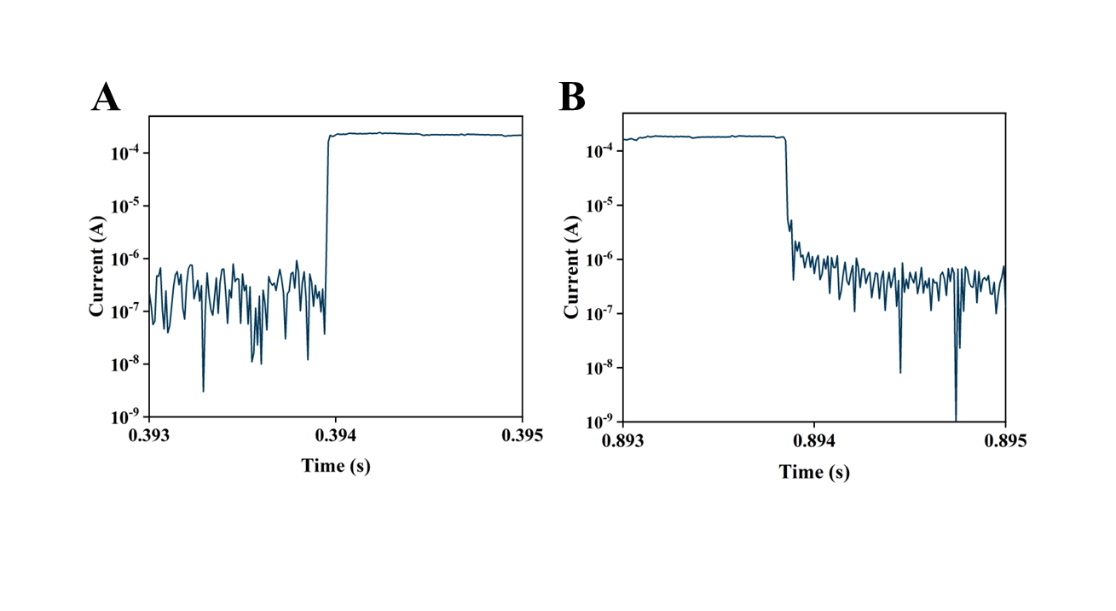
**

**FIGURE S4.** Transient response of the photodiode under 1 Hz illumination: (A) magnified rise time and (B) magnified decay time.

TABLE S1. Performance comparison of representative PbS QD-based NIR photodetectors.

| Year | Method | | λ range (nm) | Responsivity (A/W) or EQE | Detectivity (Jones) | Reference |
| --- | --- | --- | --- | --- | --- | --- |
| 2020 | Hot-inject | | 1100 | 100% | 3.20 × 1011 | S1 |
| 2021 | Hot-inject | | - | 50% | 6.70 × 1011 | S2 |
| 2022 | Hot-inject | | 1550 | 77 | 1.51 × 1011 | S3 |
| 2024 | Hot-inject | | 1060 | 55.5 A/W | 1.66 × 1013 | S4 |
| 2025 | Hot-inject | | 808 | 0.78 A/W | 4.38 × 1012 | S5 |
| 2025 | Hot-inject | | 1550 | 187 A/W | 2.81 × 1012 | S6 |
| 2025 | ICDS | photo-FETs | 1064 | 0.20 | 1.63 × 1011 | This work |
|  |  | photodiodes | **1064** | **0.21** | **1.10 × 10^11^** |  |

##

## References

Yang, W., Roggemann, M. C., Cooper, K., Buller, W., Subotic, N., Middlebrook, C., Boreman, G. D., et al. (2008). The IR antenna pair coupled sensor element and its potential application in wavefront sensing. Infrared phys. tech. 51(6), 495-504. doi:10.1016/j.infrared.2007.11.004

Jung, B. K., Woo, H. K., Shin, C., Park, T., Li, N., Lee, K. J., Oh, S. J., et al. (2022). Suppressing the dark current in quantum dot infrared photodetectors by controlling carrier statistics. Adv. Opt. Mater. 10 (2), 2101611. doi:10.1002/adom.202101611

Chen, D., Liu, Y., Xia, B., Chen, L., Yang, Y., Yang, G., et al. (2023). Passivating {100} facets of PbS colloidal quantum dots via perovskite bridges for sensitive and stable infrared photodiodes, *Adv. Funct. Mater.* 33 (1). 2210158. doi: 10.1002/adfm.202210158

Lee, D., Jeong, S., Moon, S., Yang, M., Kim, S. H., Kim, D., Baek, S. W., et al. (2024). Giant colloidal quantum dot/α-Ga_2_O_3_ heterojunction for high performance UV-Vis-IR broadband photodetector. *ACS nano* 18 (51), 34741-34749. doi:10.1021/acsnano.4c10960

Ding, J., Liu, X., Gao, Y., Dong, C., Yue, G., Tan, F. (2025). Charge carrier management via semiconducting matrix for efficient self-powered quantum dot infrared photodetectors. *Journal of Semiconductors* 46 (3), 032401. doi:10.1088/1674-4926/24100028

Pan, Z., Zhang, J., Zhang, L., Wei, H., Tang, W., Lin, L., Li, J., et al. (2025). Solution-processable phototransistor of PbS quantum dots/ZnO for highly responsive 1550 nm near infrared photodetection. *J. Colloid Interface Sci.* 700, 138313. doi:10.1016/j.jcis.2025.138313
